# Overview of clear cell renal cell carcinoma: from mechanisms to clinical

**DOI:** 10.3389/fmed.2026.1822724

**Published:** 2026-06-24

**Authors:** Han Yang, Xingda Zhang, Qingming Ji, Xia Sheng

**Affiliations:** 1 Department of Nephrology, Jilin Province People's Hospital, Changchun, Jilin, China; 2 Department of Intensive Care Medicine, The First Hospital of Jilin University, Changchun, Jilin, China; 3 Department of Clinical Nutrition, China-Japan Union Hospital of Jilin University, Changchun, China

**Keywords:** Belzutifan, cancer, clear cell renal cell carcinoma, clinical trial, HIF-2α

## Abstract

Clear cell renal cell carcinoma is the most common histological subtype of kidney cancer, and its development is often related to mutations in the VHL gene. Inactivation of the VHL gene leads to accumulation of HIF-2α. The accumulation of HIF-2α activates multiple signaling pathways, promoting tumor growth and proliferation. The treatment strategies for clear cell renal cell carcinoma mainly include surgical resection, targeted drugs, and immune checkpoint inhibitors. Previous targeted treatments have mostly been tyrosine kinase inhibitors. In recent years, the HIF-2α inhibitor Belzutifan has been approved by the FDA for the treatment of clear cell renal cell carcinoma due to its specificity in blocking the HIF-2α signaling pathway and its ability to inhibit tumor growth. This article, based on HIF-2α, provides an overview of the pathogenesis, diagnosis, and treatment of clear cell renal cell carcinoma, with a focus on the clinical trial results of Belzutifan, offering direction and information for the clinical treatment of clear cell renal cell carcinoma.

## 1 Introduction

Renal cell carcinoma is the most common type of cancer in the urinary system, accounting for 5% of all malignancies and ranking as the sixth and tenth most common cancer type in men and women, respectively (1). About 430,000 people develop renal cell carcinoma annually and it causes more than 179,000 deaths per year in worldwide (2). There are several subtypes of renal cell carcinoma, with the most common being clear cell renal cell carcinoma (ccRCC), accounting for more than 3/4 of renal cell carcinomas (3). Genetic factors are involved in ccRCC. Statistically, ccRCC almost always has mutation of VHL gene (4), and about 70% of patients with VHL disease develop ccRCC during their lifetimes (5). Loss of VHL function leads to an accumulation of hypoxia-inducible factors (HIFs), which in turn upregulate pro-tumorigenic hypoxic response genes, including the Vascular Endothelial Growth Factor A (VEGFA), the main driving force in the development of ccRCC (4, 6). Additionally, metabolic reprogramming by VHL/HIF-mediated pseudohypoxia renders ccRCC a metabolism-related disease (7, 8).

In the clinic, most kidney cancers are diagnosed incidentally, with diagnosis primarily relying on imaging tests. Early diagnosis is crucial for reducing the mortality rate of the disease. However, the optimal screening methods and strategies have not yet been determined (9). For the treatment of kidney cancer, it is generally believed that surgical intervention should be considered if the diameter of the renal tumor exceeds 3 cm or if the tumor is growing rapidly to reduce the risk of metastatic tumors (10–12). Where feasible, nephron-sparing surgery is adopted for renal tumors; for renal tumors with a diameter less than 3 cm, early surgical intervention is typically not recommended as these tumors have a lower risk of metastasis (5). Patients with ccRCC associated with VHL disease usually undergo several surgeries in their lifetime to remove renal tumors and other tumors related to VHL disease (13). Although surgical treatment is feasible, there are still 20% ~ 30% of patients who develop distant metastases, and 2 to 5% of patients experience local recurrence, so postoperative adjuvant therapy is very important. Postoperative adjuvant treatments include radiation therapy, immunotherapy, hormone therapy, vaccines, and targeted drugs. Although systemic treatments help prevent tumors from exceeding 3 cm, thereby reducing the need for surgery and metastasis, these methods have not provided any benefit to patient survival rates (14), so the development of new targets and drugs is imperative.

Based on the above background, this paper will provide an overview of the occurrence mechanism, diagnosis, treatment targets, and current status of ccRCC, with a focus on clinical drug trials importantly, in order to assist in determining diagnoses and selecting the best treatment methods for patients with ccRCC.

## 2 Mechanism of ccRCC

### 2.1 Risk factors of ccRCC

**Figure 1:**
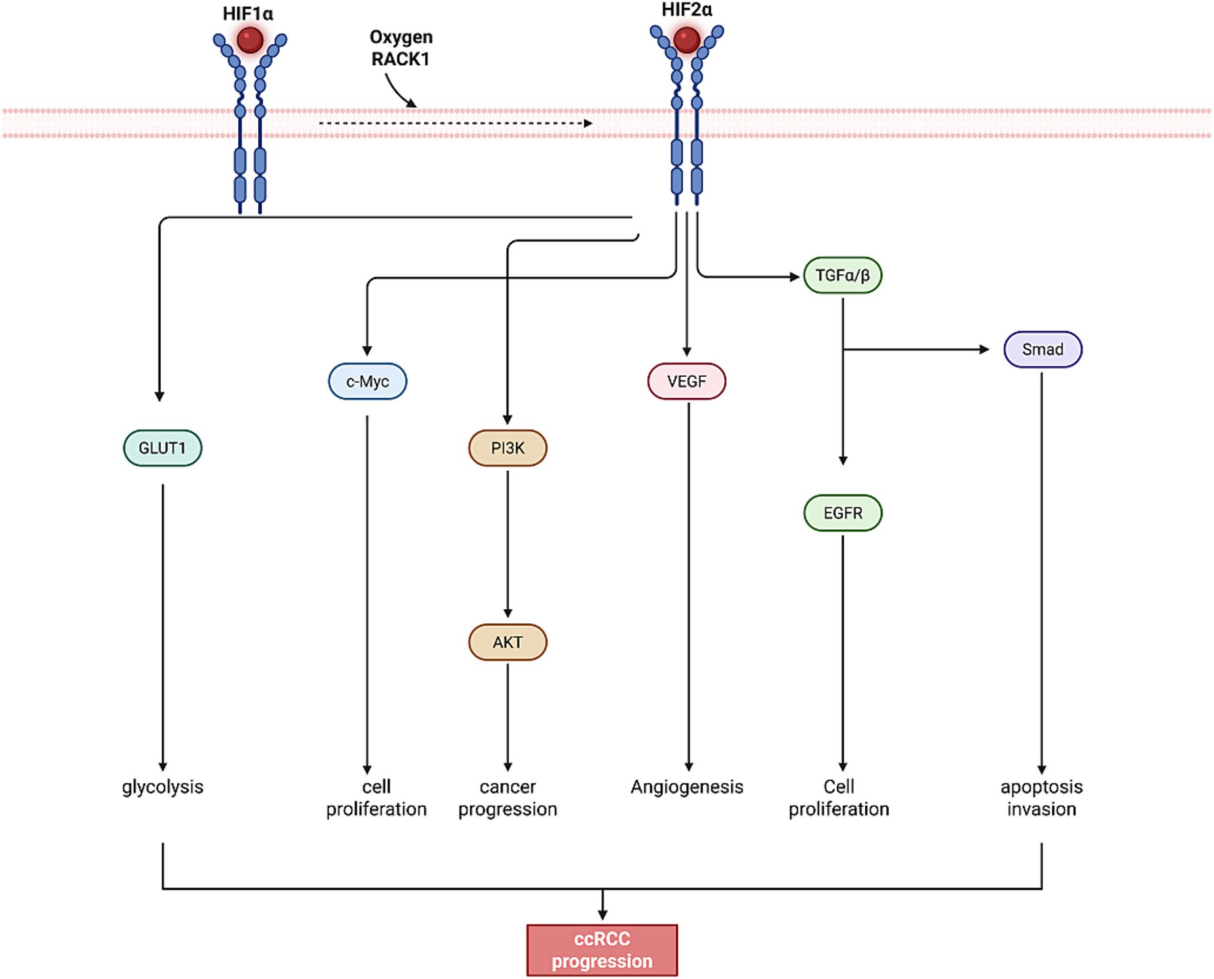
Risks factors for ccRCC.

The incidence of ccRCC increases with age and is higher in men than in women (15, 16). Genetic factors are the major cause of ccRCC. ccRCC is often associated with VHL disease, a rare autosomal dominant hereditary cancer syndrome that is associated with inactivation of a single VHL gene (mutation, deletion or hypermethylation) leading to loss of VHL function (17, 18). Established risk factors (Figure 1) that are associated with the development of ccRCC include smoking, hypertension, obesity and acquired renal disease (15, 16, 19–22). Also the family history of chronic and cystic kidney diseases, kidney transplantation, tuberous sclerosis and diabetes mellitus (15, 21, 23–25).

### 2.2 VHL in ccRCC

VHL is a tumor suppressor gene that maps to chromosome 3p25 (26). Mutations of one allele of the chromosome lead to the formation of VHL disease (16), and inactivating mutations of the remaining VHL allele are drivers of ccRCC development (27). The VHL gene can be inactivated by point mutations, insertion or deletion mutations, loss of heterozygosity of alleles at 3p25 and epigenetic alterations including hypermethylation of the VHL promoter resulting in loss of pVHL function (28–31). pVHL is an integral component of the mammalian E3 ubiquitin ligase complex and also a receptor subunit of the E3 ubiquitin ligase complex that is responsible for the ubiquitination and proteasomal degradation of a wide variety of substrates (32–35). pVHL plays an important role in oxygen sensing, regulating HIFs activity under normoxic conditions. Aerobically, HIF-1α and HIF-2*α* are continuously synthesized and degraded (36), a mechanism that ensures that HIF transcription factors accumulate only under hypoxic/anoxic conditions. So the absence of pVHL affects the stabilization and accumulation of HIF-α, which activates HIF target genes that influence a variety of pathophysiologic processes including angiogenesis, cell growth, cell proliferation, glycolysis and apoptosis (37).

### 2.3 HIF in ccRCC

Almost all pVHL mutant associated RCCs result in an accumulation of HIF-*α* and overexpression of HIF target genes (38). Initially both HIF-1α and HIF-2α accumulate, however over time HIF-2α predominates and suppresses HIF-1α expression at the protein level (39). During the initial phase of acute hypoxia, HIF-1α accumulates and functions, whereas during long-term hypoxic conditions, HIF-2α predominates, leading to a shift from HIF-1α to HIF-2α activity (40). A similar phenomenon is observed during ccRCC progression, wherein HIF-2α accumulation eventually exceeds that of HIF-1α to facilitate tumor growth and metastasis. Apart from oxygen regulation of HIFs, other mechanisms are involved in the HIF switch process. The receptor for activated protein kinase C (RACK1) competes with heat shock protein 90 (HSP90) to bind with HIF-1α, thereby promoting ubiquitination of HIF-1α and its subsequent degradation (41). MicroRNAs (miRNAs) and long noncoding RNAs are also involved in regulating the HIF-1α to HIF-2α switch. The activity of miR-18a for instance, significantly increases during prolonged hypoxia and directly targets HIF-1α mRNA, thereby causing the HIF switch (42). In mouse renal carcinoma xenograft models, HIF-2α overexpression promotes tumor growth while knockdown of HIF-2α inhibits tumor growth. Conversely, HIF-1α overexpression reduces tumor volume and HIF-1α knockdown promotes cell proliferation (38, 39, 43, 44). These results suggest that HIF-1α leans toward a tumor suppressor role, while HIF-2α is more of an oncogene. HIF-1α regulates glycolysis, whereas HIF-2α regulates genes involved in lipoprotein metabolism, ribosome biogenesis, and F2F and MYC transcriptional activity. Studies have shown that HIF1A single copy deletion or high levels of HIF2A mRNA expression correlate with alterations in immune microenvironment in ccRCC. Balanced changes in HIF-1α and HIF-2α activities affect different aspects of ccRCC biology and disease aggressiveness (45). Overexpression of HIF-2α stimulates a variety of signaling pathways, thereby contributing to ccRCC initiation and progression.

### 2.4 HIF-2*α*-driven signaling pathways in ccRCC progression and metastasis

**Figure 2:**
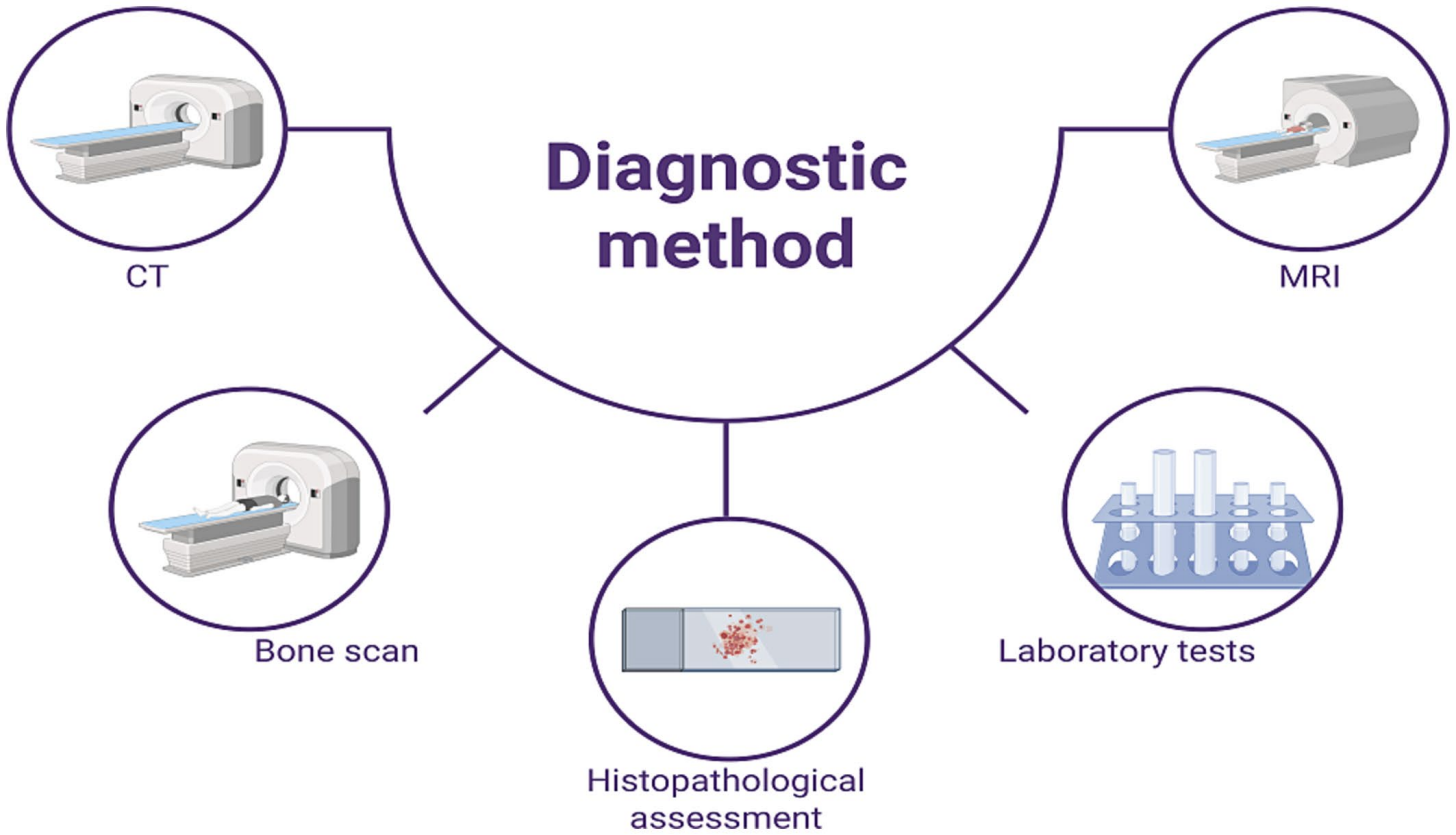
HIF2α-driven signal pathways of ccRCC.

The accumulation of HIF-2*α* caused by loss of VHL function leads to ccRCC progression and metastasis through many signal pathways (Figure 2), such as an elevation of transforming growth factor *α* (TGF-α), a ligand of the epidermal growth factor receptor (EGFR), in ccRCC patients (46). TGF-*α* promotes cell proliferation by stimulating signal transduction of EGFR, which results in ccRCC progression. And blocking the EGFR inhibits HIF-2α-mediated tumorigenesis (47–49), suggesting that HIF-2α is a major factor affecting the TGF-α/EGFR-mediated pathway of ccRCC initiation and progression.

The c-Myc is an original oncogene. Increased expression of c-Myc and its target genes in VHL-deficient ccRCC suggests its role in tumor progression (50). HIF-2α was found to elevate the activity of c-Myc promoting cell cycle progression in ccRCC. In contrast, HIF-1α suppresses growth-responsive genes of c-Myc inhibiting cell cycle progression (51, 52). Knockdown of Myc suppresses the growth of ccRCC cells, inducing a stop in the cell cycle (50). These findings suggest that upregulation of c-Myc is critical for ccRCC progression.

Hypoxia is a major regulator of angiogenesis, and abnormal angiogenesis is the key to a variety of pathological conditions including solid tumors. HIFs regulate the expression of multiple pro-angiogenic genes, among which is VEGF (53). VEGF is a potent pro-angiogenic agent. It was found that VEGFA is expressed at significantly increased levels when pVHL is inactivated in ccRCC, and VEGF overexpression correlates with the clinical stage, tumor grade, lymph node metastasis and overall survival of ccRCC (54–58). HIF-2α is an upstream regulatory factor of VEGF expression in ccRCC, and inhibition of HIF-2α by RNA interference can reduce VEGF expression (59–61).

Reprogrammed metabolism of tumor cells can meet higher demands of cells for nutrients such as glucose, amino acids, and lipids to support cell proliferation (62, 63). In ccRCC, HIF-2*α* induces the expression of glucose transporter-1 (GLUT-1) (59). Glucose deprivation or inhibition of GLUT-1 activity attenuates ccRCC progression and promotes tumor cell apoptosis (64).

In addition, CPT1A is a direct target of HIF-α and CPT1A inhibits the growth of VHL-deficient ccRCC, its expression is repressed by both HIF-1α and HIF-2α (65). PLN2 promotes ccRCC tumor growth, its expression level is upregulated by a HIF-2α dependent mechanism (66). The tyrosine kinase receptor MET is often aberrantly expressed in ccRCC, inactivation of VHL in ccRCC leads to constitutive activation of MET (67). Overexpression of MET may be an initial oncogenic event triggered by VHL deletion that promotes tumor invasion and metastasis, suggesting its potential as a downstream candidate in the context of HIF-*α* accumulation (68, 69). AXL, also a tyrosine kinase receptor, is a direct target of HIF-2α, its overexpression is associated with ccRCC invasion and metastasis (70). Membrane type 1-matrix metalloproteinase (MT1-MMP) expression is induced by HIF-2α stabilization in VHL-inactivated ccRCC, and MT1-MMP overexpression is associated with ccRCC aggressiveness (71, 72). PI3K/AKT/mTOR signaling controls cell growth differentiation, migration, angiogenesis and so on. Overactivity of mTOR signaling is frequently observed in VHL-inactivated ccRCC and is associated with tumor aggressiveness and poor prognosis (73, 74). In addition, mTORC 1 activity is modulated by a HIF-2α-dependent mechanism. Silence of HIF-2α inhibits mTORC 1 activity and reduces tumor size, which is consistent with the pro-tumorigenic properties of HIF-2α (75). Cyclin D1 (encoded by the CCND1 gene) is upregulated upon inactivation of VHL, whereas reintroduction of pVHL decreases the level of cyclin D1 (76, 77). Knockdown of CCND1 can reduce the xenograft tumor growth derived from VHL-null ccRCC (59). This result is consistent with those showing that HIF-2α maintains tumor growth though cyclin D1 activation in VHL-deficient renal cell carcinoma (38, 43).

## 3 Diagnosis of ccRCC

**Figure 3:**
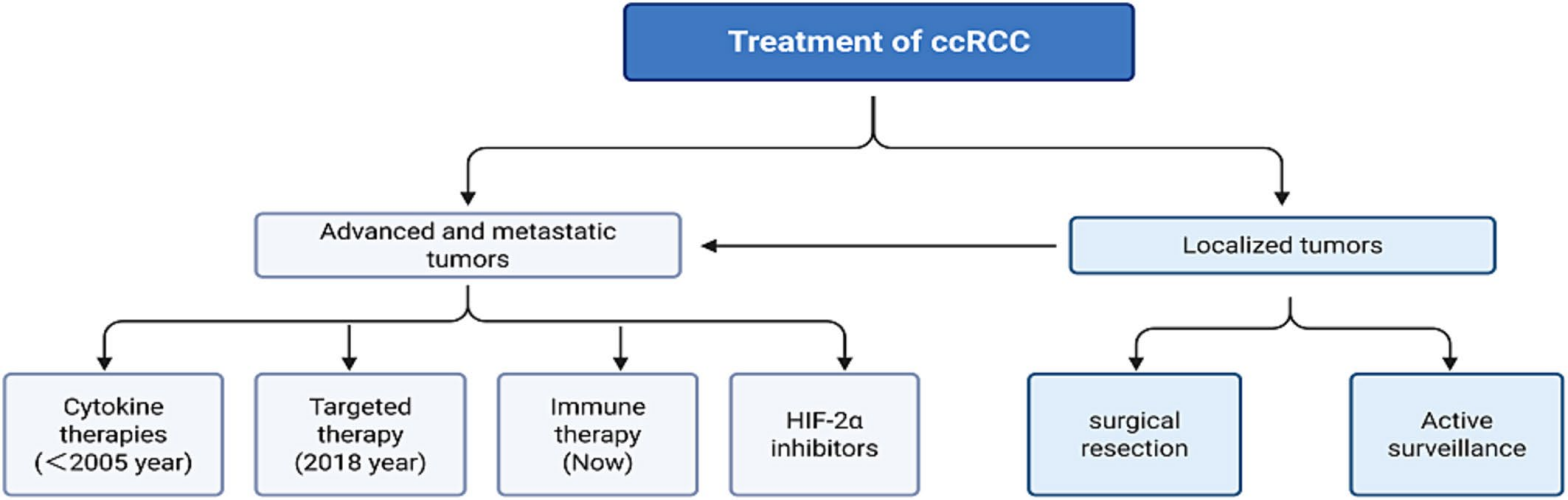
Diagnosis of ccRCC.

Initial diagnosis of ccRCC is classically based on the typical presentation: flank pain, gross hematuria and palpable abdominal mass. This diagnostic approach has been largely superseded. For all stages of ccRCC, contrast-enhanced CT of the chest, abdomen and pelvis is performed for assessment of tumor stage. For advanced or metastatic tumors, CT or MRI and bone scan should be performed before starting systemic therapy. PET-CT is not recommended for routine ccRCC staging or restaging. In addition to imaging studies, histopathological assessment is mandatory before starting systemic therapy in all patients. Biopsy of the tumor or metastasis or intraoperative examination of resected samples provides a sensitive and specific pathological diagnosis without the risk of tumor dissemination (78, 79). Histopathological assessment determines the subtype and the presence of sarcomatoid or rhabdoid differentiation, which are of prognostic and therapeutic significance, respectively (80). Furthermore, laboratory tests including serum creatinine, hemoglobin, leukocyte and platelet count, lymphocyte-to-neutrophil ratio are also used for the prognosis evaluation and treatment selection for advanced disease (81)(Figure 3).

## 4 Treatment of ccRCC

**Figure 4:**
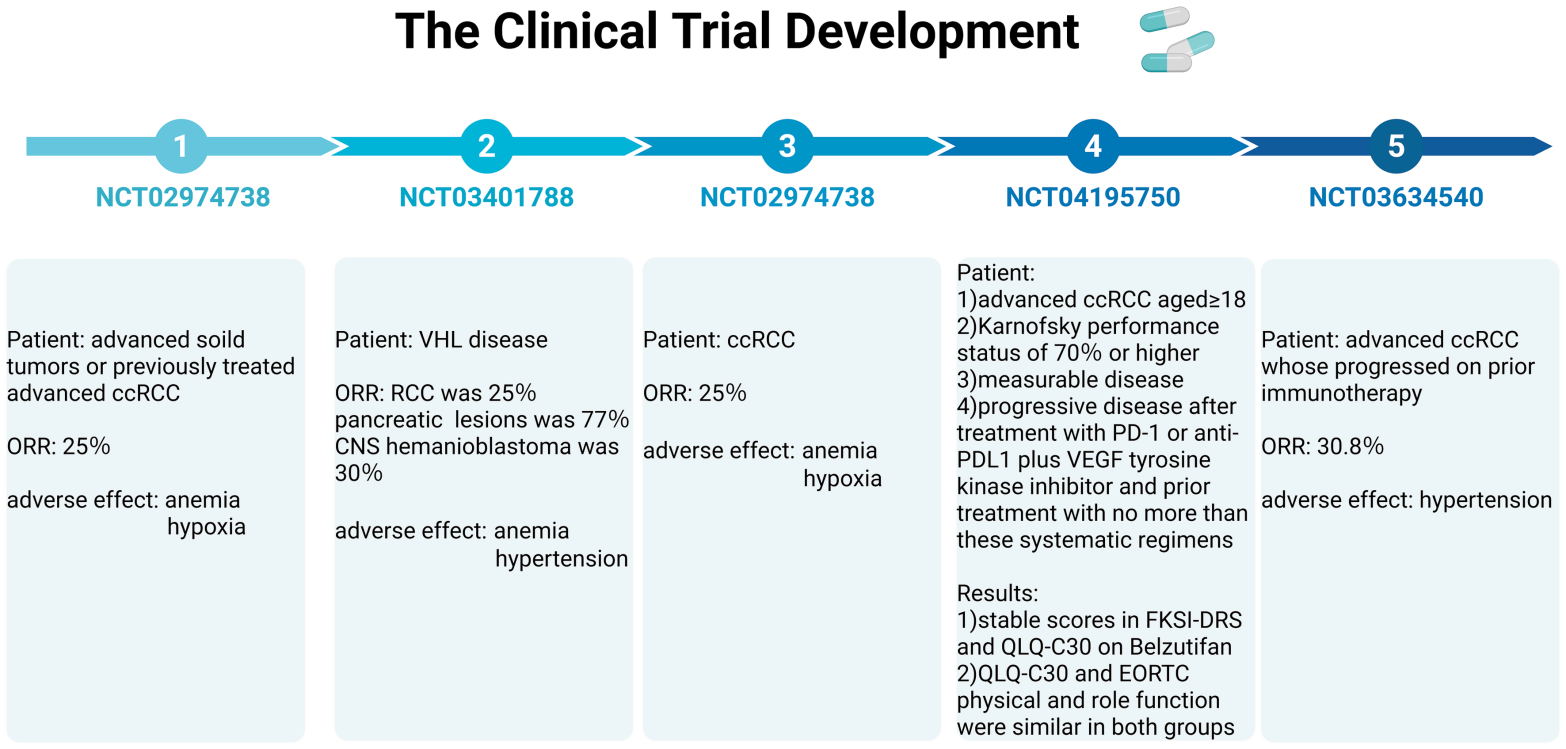
Treatment of ccRCC.

The majority of ccRCC patients have localized tumors at the time of diagnosis. For patients with localized tumors, the standard treatment is surgical resection, including radical nephrectomy or partial nephrectomy. Active surveillance with or without tumor biopsy or tumor ablation is also a choice for eligible patients. Clinically, recurrence or progression still occurs in a proportion of patients despite definitive local treatment, with a 5-year recurrence-free survival of approximately 42% ~ 98% (20). Patients with advanced and metastatic ccRCC are usually required to undergo systemic therapies. Before 2005, patients with metastatic ccRCC had limited treatment options other than cytokine therapies, including interferon and IL-2. With the improved understanding of the pathogenesis of the disease, targeted therapy as first-line treatment until 2018, mainly targeting VEGFR (sunitinib, pazopanib, sorafenib, axitinib, cabozantinib, lenvatinib and other CEGF-TKIs) and VEGF (bevacizumab) as well as mTOR pathway (everolimus and temsirolimus) targeted drugs that have been approved by the FDA for the treatment of RCC patients (82–87). Currently immuno-checkpoint inhibitor based doublets have resulted in significant improvement in overall survival in patients with metastatic tumors and have become the new standard of care. The immuno-checkpoint inhibitor based doublets are divided into two categories: (1) dual ICI combinations that target both CTLA-4 and PD-1, i.e., ICI-ICI; and (2) combination of anti-PD1 ICI with VEGF tyrosine kinase inhibitors, i.e., ICI-TKI combinations (88–91). The combination treatment mode is mainly using antiangiogenic drugs, however, antiangiogenic therapy has limited efficacy in cancer. Because tumor angiogenesis is a complex process that varies from one tumor type and anatomical location to another (92)(Figure 4). Furthermore, many patients exhibit poor response to antiangiogenic drug therapy and have certain side effects, which may be avoidable by increasing the number of new targetable genes and pathways.

Mutation in pVHL leads to HIF-2α accumulation which drives several pathways favorable for growth and proliferation, metastasis of ccRCC, moreover, in 2009, Scheuermann et al., identified a pocket in the specific domain of HIF-2α protein which can accommodate small molecules and has the potential to be targeted by inhibitors that disrupt heterodimerization of HIF-2α protein complex. This discovery led to the development of HIF-2α inhibitors (93). Belzutifan (MK-6482 or PT2977) is a small molecule HIF-2α inhibitor which selectively disrupts the heterodimerization of HIF-2α with HIF-1β (94). The FDA recently approved Belzutifan for patients with VHL disease-related tumors such as renal cell carcinomas (patients with localized disease), central nervous system (CNS) hemangioblastoma or pancreatic neuroendocrine neoplasms (95). But the development of resistance is a major reason for the low survival rate in ccRCC patients. Further research targeting HIF-2α signaling network might identify novel therapeutic targets, develop new therapeutic agents providing novel therapeutic avenues favoring disease management.

## 5 Clinical trials of HIF-2α-related drugs in ccRCC

**Figure 5:**
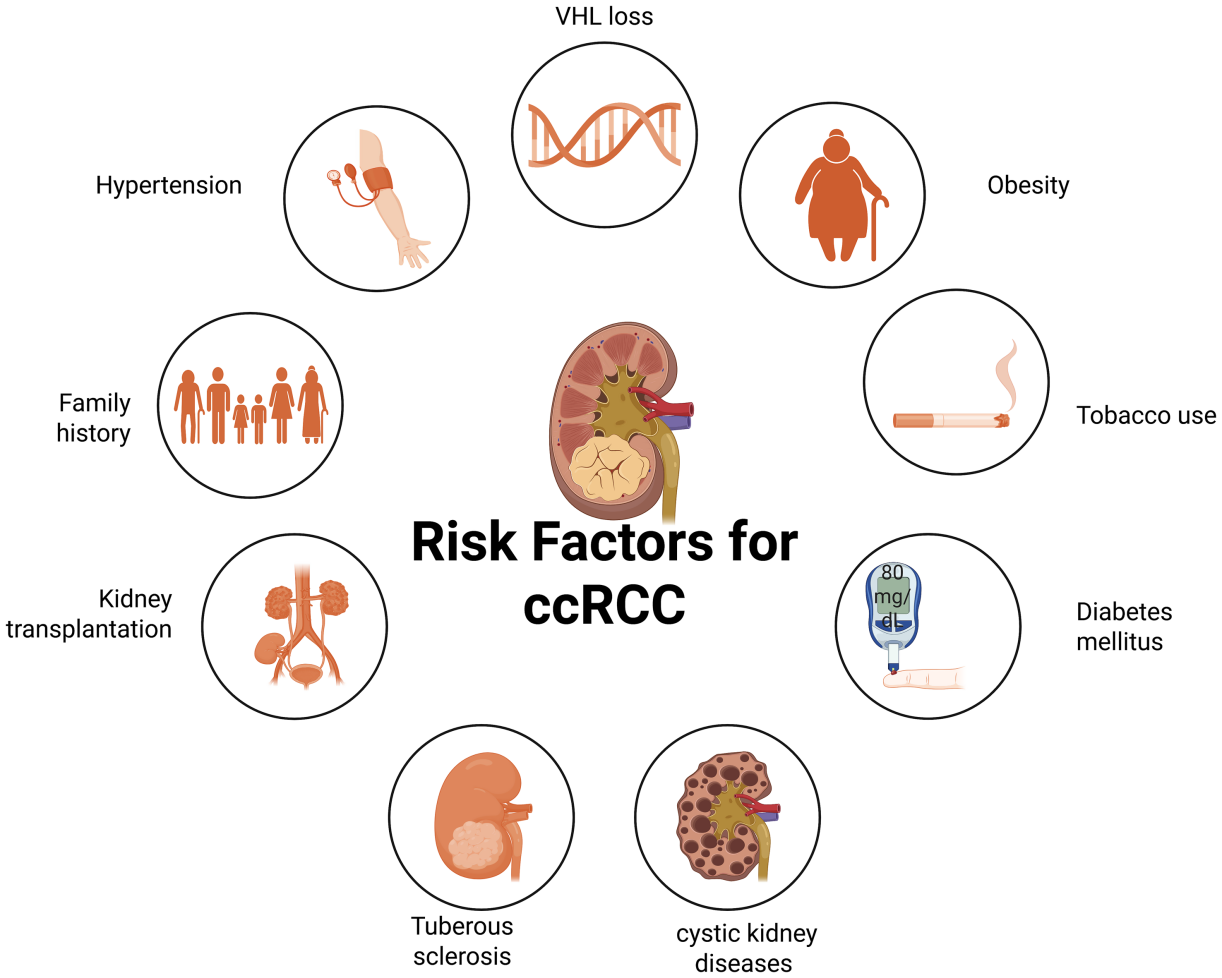
Development of Belzutifan clinical trials.

VHL disease is the most common hereditary RCC syndrome, resulting in the accumulation of HIF-2α, which in turn promotes the development of RCC. FDA approved Belzutifan is the first treatment targeting the VHL gene pathway, a small-molecule inhibitor of HIF-2α involved in transcription of several genes. Several clinical trials (Figure 5) have reported activity data for Belzutifan. In 2021, the FDA approved Belzutifan for patients with VHL disease with RCC (96). Also approved for patients with VHL disease-associated CNS hemangioblastoma or pancreatic neuroendocrine neoplasm (95). In late 2023, the FDA approved Belzutifan for patients with advanced RCC who had received prior PD-1 or PD-L1 and VEGF-TKI treatments (97).

In 2021, a phase 1 clinical trial (NCT02974738) of Bezultifan for the treatment of ccRCC was published. The study aimed to assess the maximum tolerated dose, safety, pharmacokinetics, pharmacodynamics, and antitumor activity of Belzutifan. Patients with advanced solid tumors (dose-escalation cohort) or previously treated advanced ccRCC (dose-expansion cohort) were enrolled. Belzutifan was administered orally in a 3 + 3 dose-escalation manner, and subsequently expanded at the recommended phase 2 dose (RP2D) in ccRCC patients. Results found that no dose-limiting toxicities were observed up to 160 mg once daily and the maximum tolerated dose was not reached in the dose-escalation cohort (*n* = 43); and the RP2D was 120 mg once daily. Decreases in plasma erythropoietin (EPO) were observed across all dose groups, and the EPO concentrations were correlated with Belzutifan plasma concentrations. Among ccRCC patients who received 120 mg once daily (*n* = 55), the confirmed objective response rate was 25% (all partial responses), and the median progression-free survival was 14.5 months. The most common grade≥3 adverse events were anemia (27%) and hypoxia (16%). Belzutifan was well tolerated and showed preliminary antitumor activity in heavily pretreated patients, suggesting that HIF-2α may be a potent target for the treatment of ccRCC (98).

A multicenter, open-label, single-arm phase 2 clinical trial (NCT03401788) published in 2022 aimed to evaluate the efficacy and safety of Belzutifan in VHL disease-related tumors. A total of 61 confirmed VHL disease patients were enrolled and administered 120 mg Beltizufan orally daily, with the primary endpoint being the objective response rate (ORR, RECIST 1.1 criteria) of ccRCC, and secondary endpoints including the response rate of non-ccRCC tumors and safety, among others. The results showed that after a median follow-up of 21.8 months, the objective response rate for patients with renal cell carcinoma was 49% (95% CI, 36–62). The response rate for patients with pancreatic lesions was 77% (47/61), and for patients with CNS hemangioblastoma was 30% (15/50). Among 12 evaluable patients with retinal hemangioblastoma, all 16 eyes improved. Grade ≥3 adverse events were mainly anemia and hypertension. Seven patients discontinued the drug: 4 voluntarily, 1 due to dizziness, 1 due to investigator-assessed disease progression, and 1 due to acute fentanyl toxicity resulting in death. The trial suggests that Belzutifan demonstrates 49% response rate and good safety in VHL disease-related renal cancer, pancreatic neuroendocrine tumors, and hemangioblastoma (96). Based on this clinical trial, the FDA approved Belzutifan for patients with VHL disease renal cell carcinoma.

LITESPAKE-001 (NCT02974738) is an open-label study with a 3 + 3 dose escalation design followed by expansion phase. Patients with ccRCC enrolled in 7 centers received Belzutifan 120 mg once daily until progression, unacceptable toxicity or patient withdrawal. The primary endpoint is to determine the maximum tolerated dose and/or recommended phase II dose. Secondary endpoints include objective response rate and duration of response assessed by investigator according to Response Evaluation Criteria In Solid Tumors (RECIST) 1.1 criteria and safety. The median follow-up time for this trial is 41.2 (38.2 ~ 47.7) months, patients had received a median of 3 (1 ~ 9) prior system therapies. The results are as follows: among 55 patients, 14 (25%) achieved objective response. The median duration of response was not reached (range, 3.1 + to 38.0 + months). 53 (96%) reported investigator-assessed adverse events related to study treatment. Grade 3 treatment-related adverse events occurred in 22 (40%) patients; the most common adverse events were anemia and hypoxia. There were no grade 4 or 5 treatment-related adverse events. The results suggest that Belzutifan monotherapy shows durable antitumor activity with an acceptable safety profile in advanced ccRCC (94). The results of this trial support the development of subsequent trials and combination strategies.

LITESPAKE-005 (NCT04195750) was an open-label, multicenter, randomized, active-controlled phase III trial conducted at 147 hospitals and cancer centers in 6 regions. Eligibility criteria: patients with advanced ccRCC aged ≥ 18 years, Karnofsky Performance Status of 70% or higher, measurable disease according to RECIST version 1.1, progressive disease after treatment with PD-1 or anti-PD-L1 immunotherapy plus VEGF tyrosine kinase inhibitor and prior treatment with no more than three systemic regimens. Seven hundred forty-six participants meeting the eligibility criteria were randomized to receive Belzutifan (*n* = 374) or everolimus (*n* = 372) once daily oral 120 mg Belzutifan or once daily oral 10 mg everolimus, respectively. Primary endpoints were progression-free survival and overall survival. Patients reported outcomes (PRO) prespecified as secondary in the LITESPARK-005 study were evaluated using the Functional Assessment of Cancer Therapy—Kidney Cancer Symptom Index: Disease-Related Symptoms (FKSI-DRS) and the European Organization for Research and Treatment of Cancer Quality of Life Questionnaire Core 30 (EORTC QLQ-C30). The PRO analysis population included all participants who received at least one dose of study treatment and completed at least one PRO assessment. A constrained longitudinal data analysis model was used to evaluate the change from baseline to Week 17 in PRO least squares mean (LSMean). The time to worsening of physical and role functioning was evaluated in the PRO analysis population. The PRO full analysis set population included 366 participants for Belzutifan and 354 participants for the everolimus group. The median time from randomization to the data cutoff date of the database (June 13, 2023) was 25.7 months (interquartile range 21.7–30.4). The completion rates of the FKSI-DRS and QLQ-C30 at baseline were both above 94% and at Week 17 were above 55% in each group. Changes in FKSI-DRS scores from baseline to Week 17 (between-group LSMean difference 1.5 [95% CI 0.7 to 2.2]) and QLQ-C30 scores (6.4 [3.2 to 9.6]) from baseline demonstrated stable scores for patients on Belzutifan versus a decrease in the everolimus group. Changes in scores on the QLQ-C30 Physical Functioning (between-group LSMean difference 2.5 [95% CI - 0.6 to 5.5]) and QLQ-C30 Role Functioning (4.2 [0.1 to 8.4]) subscales from baseline to Week 17 were similar in both groups. EORTC Physical Functioning (Median 19. The time to deterioration in overall survival (median 11.1 months [95% CI 11.1 to not reached] vs. 13.8 months [10.6 to not reached] for the bezutifan group; HR 0.93 [95% CI 0.72 to 1.20]) and role functioning (median 12.0 months [9.2 to not reached] vs. 10.2 months [4.7 to 14.4]; 0.88 [0.69 to 1.11]) were similar between the bezutifan and the envision groups. Belzutifan treatment of advanced RCC improves disease-specific symptoms and QoL compared with envision. Combining efficacy and safety data from the LITESPARK-005 study, bezutifan provides clinical benefit to patients in this treatment setting without compromising their QoL (99). Based on this, the FDA approved bezutifan for patients with advanced RCC who have progressed on PD-1 or PD-L1 and VEGF-TKI. There are corresponding clinical trials to study the effectiveness of Beltizufan in combination therapy afterwards.

LITESPARK-003 (NCT03634540) is an open-label, single-arm, phase II study to evaluate the antitumor activity and safety of Belzutifan combined with Cabozantinib in patients with advanced ccRCC whose disease had progressed on prior immunotherapy. 52 patients received at least one dose of treatment, who were 18 years of age or older with locally advanced or metastatic ccRCC. Once daily oral doses of 120 mg Bezutifan and 60 mg Cabozantinib were administered, with the primary endpoint being the investigator-assessed objective response rate. Antitumor activity and safety were assessed among all patients who received at least one dose of study treatment. The median follow-up time was 24.6 months. The results showed that the patient ORR was 30.8% (95% CI 18.7–45.1), with 2% being complete responses and 29% being partial responses. The most common grade 3 ~ 4 treatment-related adverse event was hypertension (27%). Severe treatment-related adverse events occurred in 15 (29%) patients. One death was considered treatment-related (respiratory failure). The trial showed that Belzutifan combined with Cabozantinib demonstrated promising antitumor activity in patients with previously treated ccRCC (100), and the findings could provide a basis for further trials of belzutifan combination therapy.

In addition to belzutifan, another novel HIF-2α inhibitor, DFF332, conducted a phase I, open-label, multicenter, dose-escalation study (NCT04895748) in patients with locally advanced or metastatic ccRCC who had previously received immunotherapy and VEGF-targeted therapy. However, the objective response rate was only 5% (101). This suggests its preliminary anti-tumor activity is limited, recommending subsequent optimization of experimental protocols or combination therapies.

## 6 Conclusion and perspectives

Renal cell carcinoma is an important part of cancer, especially ccRCC. ccRCC is considered have a close relationship with gene-VHL. This article reviewed the mechanism, diagnosis, treatment and its limit, clinical trials of ccRCC, provide a guideline for clinical treatment. Now many strategies have been suggested effectively in the treatment of ccRCC. But there are still have amounts of questions, such as low response rate, poor tolerance and large adverse effects of ICIs and TKIs. This driven the drugs development of ccRCC. At present, the research on HIF-2α inhibitor Belzutifan is extensive. Clinical trials have shown good efficacy and safety. Belzutifan has clinical translational value. Compared with traditional TKIs and immunotherapy, HIF-2safety. d safety. Own good efficacy and safety. y. y and safety. Ety. hibitor Belzutifan is extExcept Belzutifan, hoping develop more HIFs inhibitors to resolve current predicament. Future research should focus on the application of combination therapies, such as integration with immunotherapy; however, broader preclinical studies are required. Effective clinical translation necessitates rigorous trial design encompassing pharmacodynamic evaluations, biomarker-based patient selection strategies, and comprehensive safety monitoring protocols. We also can use nanotechnology or AI to achieve our goal.
